# CRISPR/Cas9‐RNA interference system for combinatorial metabolic engineering of Saccharomyces cerevisiae

**DOI:** 10.1002/yea.3390

**Published:** 2019-06-13

**Authors:** Kanchana Rueksomtawin Kildegaard, Larissa Ribeiro Ramos Tramontin, Ksenia Chekina, Mingji Li, Tobias Justus Goedecke, Mette Kristensen, Irina Borodina

**Affiliations:** ^1^ The Novo Nordisk Foundation Center for Biosustainability Technical University of Denmark Kongens Lyngby Denmark

**Keywords:** *cis,cis*‐muconic acid, CRISPR/Cas9, genome editing, metabolic engineering, RNA interference, USER (uracil‐specific excision reagent), Saccharomyces cerevisiae

## Abstract

The yeast Saccharomyces cerevisiae is widely used in industrial biotechnology for the production of fuels, chemicals, food ingredients, food and beverages, and pharmaceuticals. To obtain high‐performing strains for such bioprocesses, it is often necessary to test tens or even hundreds of metabolic engineering targets, preferably in combinations, to account for synergistic and antagonistic effects. Here, we present a method that allows simultaneous perturbation of multiple selected genetic targets by combining the advantage of CRISPR/Cas9, *in vivo* recombination, USER assembly and RNA interference. CRISPR/Cas9 introduces a double‐strand break in a specific genomic region, where multiexpression constructs combined with the knockdown constructs are simultaneously integrated by homologous recombination.

We show the applicability of the method by improving *cis*,*cis*‐muconic acid production in S. cerevisiae through simultaneous manipulation of several metabolic engineering targets.

The method can accelerate metabolic engineering efforts for the construction of future cell factories.

## 1 INTRODUCTION

Industrial biotechnology uses cell factories to produce therapeutical proteins, antibiotics, enzymes, fuels, and chemicals. To achieve favorable process economics, one needs to optimize the cell factories, where performance metrics as titer, rate, and yield are improved. Strain development programs for the products that are not native to the host are very costly and take a long time. The required investment in biotechnology companies that develop novel strains and processes is typically above $50 Mio. During the strain development, hundreds to thousands of strain variants are engineered in iterative design‐build‐test cycles. High‐throughput strain construction and screening in the range of 10^5^–10^6^variants are possible when a biosensor indicating the product presence is available (Zhang, Jensen, & Keasling, 2015); however, this is seldom the case. Hence, the main course of action remains laborious manual strain construction via polymerase chain reaction (PCR), cloning, and transformations. The cloning and strain construction is typically performed at 10–50‐μl scale, where the high cost of specialized reagents also contributes to the high price of the strain development.

Metabolic engineering research requires tools for multiplex genome editing that would allow simultaneous upregulation and downregulation of multiple genes in a combinatorial way. Clustered regularly interspaced short palindromic repeats system with associated nuclease Cas9 (CRISPR/Cas9) system has dramatically simplified genome editing in yeasts, particularly for performing gene overexpression, mutations, and deletions (Lian, HamediRad, & Zhao, 2018; Stovicek, Holkenbrink, & Borodina, 2017). Convenient CRIPSR/Cas‐based genetic tools have been developed for Saccharomyces cerevisiae that enable integration of several gene expression cassettes into multiple locior simultaneous deletion of multiple genes in a single transformation (Bao et al., 2014; Generoso, Gottardi, Oreb, & Boles, 2016; Horwitz et al., 2015; Jakočiūnas et al., 2015; Ryan et al., 2014; Verwaal, Buiting‐Wiessenhaan, Dalhuijsen, & Roubos, 2018). The CRISPR/Cas systems are efficient in editing not only haploid laboratory strains but also diploid and polyploid strains of S. cerevisiae important for brewing and bioethanol applications (Denby et al., 2018; Lian, Bao, Hu, & Zhao, 2018; Stovicek, Borodina, & Forster, 2015). It has also been illustrated in multiple studies how overexpressions, deletions, and mutations can be performed in a single transformation (Jakočiu̅nas et al., 2015; Lian, HamediRad, Hu, & Zhao, 2017; Mans et al., 2015).

Controlled downregulation of gene expression, however, remains a challenge. Gene downregulation is often a more desirable metabolic engineering strategy than complete gene inactivation, and, in case of essential genes, the only option. Catalytically inactivated dCas9, also in a variant coupled to a transcriptional repressor, has been applied for downregulation, but typically multiple gRNA binding sites need to be tested to obtain the desired repression level (Deaner & Alper, 2017; Jensen et al., 2017; Zalatan et al., 2015). Alternatively, RNA interference (RNAi) has been demonstrated to allow more precise control of gene downregulation (Crook, Schmitz, & Alper, 2013; Drinnenberg et al., 2009; Si, Luo, Bao, & Zhao, 2014; Suk et al., 2011).

In this study, we aimed to develop a method that would allow multiplex upregulation and downregulation of several genes by combining the advantages of the CRISPR/Cas9 system and RNAi. The level of upregulation and downregulation can be tuned by selecting promoters of different strengths. To illustrate the applicability of the method, we optimized the cell for production of a prospective chemical molecule *cis,cis*‐muconic acid (CCM).

## 2 MATERIALS AND METHODS

### 2.1 Strains, media, and chemicals

S. cerevisiae CEN.PK strains used in this study are listed in Table S1. The strain of *Naumovozyma castellii* CLIB290 was received from Centre International de Ressources Microbiennes, Institut National de la Recherche Agronomique (INRA), France. Yeast strains were grown in synthetic complete (SC) medium, synthetic drop‐out (SD) medium, defined mineral medium or synthetic fed‐batch medium Sc.syn‐1000 (M2P labs GmbH, Germany) at 30°C. SC and SD media and agar plates were prepared using premixed drop‐out powders from Sigma‐Aldrich. The defined mineral medium was prepared as described previously (Jensen et al., 2014). Escherichia coli strain DH5α was used as a host for plasmid propagation. E. coli cells were grown at 37°C in Luria–Bertani medium containing 100 μg ml^−1^ ampicillin. The chemicals were obtained, if not indicated otherwise, from Sigma‐Aldrich. Nourseothricin was obtained from WERNER BioAgents GmbH (Germany). Phusion U Hot Start DNA polymerase and PhusionHot Start II DNA polymerase were purchased from Thermo Fisher Scientific.

### 2.2 Biobricks amplification and plasmids construction

The oligonucleotides, biobricks, and plasmids used in this study are listed in Tables S2, S3, S4, and S5, respectively. Oligonucleotides were synthesized by Integrated DNA Technologies, Inc (Leuven, Belgium).

A plasmid containing Cas9 and gRNA plasmid for targeting CAN1.Y locus was obtained from Addgene (DiCarlo et al., 2013).

The genes *AGO1* and *DCR1* that encode correspondingly for the Argonaute and Dicer proteins were amplified from genomic DNA of *Naumovozyma castellii*. The genes, encoding *Klebsiella pneumoniae KpAroY.B* (AAY57854.1), *KpAroY.D* (AAY57856.1), *KpAroY.Ciso* ([BAH20873.1], *Candida albicans CaCatA* (XP_722784.1), and *Podospora anserina PaAroZ* (XP_001905369) were synthesized by GeneArt (Life Technologies) in versions codon‐optimized for S. cerevisiae. *KpAroY.B* and *KpAroY.D* encode B and D subunits of the protocatechuic acid decarboxylase (PCA‐DC), whereas *KpAroY.Ciso* encodes an isoform of subunit C of PCA‐DC. *CaCatA* encodes the catechol 1,2‐dioxygenase (CDO), and *PaAroZ* encodes the dehydroshikimate dehydratase (3‐DHDS). Plasmids expressing *CaCatA*, *PaAroZ*, *KpAroY. B*, *KpAroY.D*, and *KpAroY. Ciso* were previously constructed and described in Skjoedt et al. (2016). *TKL1* encodes the enzyme transketolase from S. cerevisiae. *ZWF1* and *ARO1* ^*∆aroE*^ genes were from S. cerevisiae. The *S. cerevisiae aro4* ^K229L^ encoded a feedback‐resistant 3‐deoxy‐D‐arabino‐heptulosonate‐7‐phosphate (DAHP) synthase with an amino acid change Aro4p^K229L^. The gene was as described in Rodriguez, Kildegaard, Li, Borodina, and Nielsen (2015).

All DNA fragments (Table S4) were amplified by PCR using Phusion U Hot Start DNA Polymerase (Thermo Fisher Scientific) with primers containing suitable overhangs for USER‐cloning and templates as described in Tables S2 and S3. The amplified products were cloned along with strong constitutive promoters into EasyClone integrative plasmids by USER cloning (Jensen et al., 2014). DNA manipulations in E. coli were carried out according to standard procedures. The clones with correct inserts were identified by colony PCR, and the plasmids were isolated from overnight E. coli cultures and confirmed by sequencing. The list of the constructed vectors can be found in Table S5.

For the construction of overexpression cassettes for *in vivo* assembly, there are five part types in our assembly standard (promoters, genes, terminators, upstream homology arm, and downstream homology arm). The specific overhangs flanking individual parts were designed and introduced at 5′ end of the forward and reverse primers as described in Table S3. All DNA parts were PCR amplified using Phusion U DNA polymerase according to the manufacturer's instructions. DNA fragments were gel purified and were assembled by consecutive procedures of USER reaction, T4 ligation, and PCR amplification of the assembled expression cassettes as follows: 17 μl of gel‐purified DNA fragments containing similar molar ratio of all parts was mixed with 2 μl of CutSmartTM buffer and 1 μl of USER enzyme (New England BioLabs). The mixes were incubated for 25 min at 37°C followed by 10 min at 25°C. After USER reaction was complete, 1 μl of T4 ligase, 3 μl of ligase buffer, and 6 μl of water were added. The mix was incubated for 5 min at room temperature. Two to three microliter of this ligation mix were used as a template for the final PCR reaction in order to amplify the whole expression cassette. The fragments were purified from the gel and used for yeast transformation (0.7 pmoles per transformation). For fragments smaller than 500 bp, ca. 2 pmoles of the fragment were used per transformation.

### 2.3 Construction of shRNAs

The small hairpin RNA (shRNA) constructs were composed of two DNA fragments. The first fragment contained approximately 250 bp sense sequence of the target gene under the control of the constitutive promoter and an 81‐bp sequence spanning intron 1 from *Schizosaccharomyces pombe rad9*. The second fragment contained the antisense sequence of the target gene together with terminator and an 81‐bp sequence of intron 1 from *S. pombe rad9*. Sense, anti‐sense, promoter, and terminator fragments were amplified by PCR. The corresponding fragments for generating sense and antisense cassettes were assembled via USER‐ligation‐PCR as described above. The intron sequence was implemented in the primer overhang.

Sense and antisense DNA fragments were introduced together with UP‐ and DW‐fragments for *CAN‐1* and were assembled into the genome of S. cerevisiae at *CAN‐1* locus via homologous recombination.

### 2.4 Construction of dsRNA

To generate double‐stranded RNA (dsRNA) constructs, the target gene was PCR amplified and assembled with *PGK1p* and *TEF1p* promoters, and *ADH1t* and *RPM9t* terminators in convergent direction via USER‐ligation‐PCR as above.

### 2.5 Yeast strains construction

All strains used in this study are listed in Table S1. The integrative plasmids were *Not*I‐linearized and transformed into S. cerevisiae cells using the lithium acetate protocol (Gietz & Woods, 2002). The cells were selected on SD medium selecting for *URA*, *HIS, LEU* and *TRP* markers. For the selection of strains carrying KanMXsyn and CloNatMXsyn, the ammonium sulfate in the SD medium was replaced with 1 g L^−1^ monosodium glutamate. The medium was supplemented with 200 μg ml^−1^ G418 sulfate and 100 μg ml^−1^ nourseothricin. The correct transformants were confirmed by PCR using primers described in Supplementary Table S2.

### 2.6 Single cell measurements of fluorescence

Colonies of S. cerevisiae strains to be tested were inoculated into 24 deep‐well plates (EnzyScreen, NL) containing 2‐ml SC medium at 30°C with 300 rpm. After approximately 24 hr, the cells were harvested and washed twice with water. The cell pellet was resuspended in 1 ml of phosphate‐buffered saline buffer. Cells were analyzed on BD FACSAria equipped with three solid‐state diode lasers: air‐cooled Coherent™ Sapphire™ solid‐state diode laser (488 nm, 100 mW), air‐cooled Coherent™ Yellow Green laser (561 nm, 100 mW), and an air‐cooled Coherent™ Deep Blue laser (445 nm, 50 mW). The following filters were used: FITC‐A, PE‐Cy5‐A, and mCFP‐A for the analysis of emission from yellow fluorescent proteins (YFP), red fluorescent proteins (RFP), and cyan fluorescent proteins (CFP), respectively. Compensation was performed according to the manufacturer's protocol (BD FACSAria II User's Guide).

Flow cytometry data were analyzed and interpreted using FlowJo software.

### 2.7 Muconic acid production in S. cerevisiae

At least 12 single colonies of each transformant were cultivated in 24‐well plate with air‐penetrable lids (EnzyScreen, NL) to test for the production of CCM. The colonies were inoculated in 1‐ml SD medium without uracil, histidine, and leucine and grown at 30°C with 250 rpm agitation at 5‐cm orbit cast for 24 hr; 300 μl of the overnight culture was used to inoculate 3 ml of defined mineral medium (pH 6.0) in 24‐deep well plate and incubated for 72 hr at the same conditions as above. Experiments were done in triplicates. At the end of the cultivation, OD_600_ was measured in microplate reader BioTek Synergy MX (BioTek). The culture broth was spun down at 3,500 *g*, and the supernatant was analyzed for CCM concentration using High‐performance liquid chromatography (HPLC).

### 2.8 Quantification of CCM and its intermediates by HPLC

The samples were diluted five times with water and then analyzed for 45 min using Aminex HPX‐87H ion exclusion column with eluent 1‐mM H_2_SO_4_ flow of 0.6 ml min^−1^. The temperature of the column was 60°C. The UV detector (Dionex) was used for detection of CCM (250 nm), PCA (220 nm), and catechol (220 nm). CCM, PCA, and catechol concentrations were quantified by comparison with the standard calibration curve.

### 2.9 qRT‐PCR analysis

The expression level of *ZWF1* in recombinant yeast strains was determined by quantitative real‐time PCR (qRT‐PCR). Samples for RNA isolation were taken from the cells grown in the mineral medium for 24 hr in triplicates. Sampling procedure and total RNA extraction were performed as previously described (Kildegaard et al., 2014). The first strand cDNA synthesis was performed using Oligo (dt)_12–18_ Primer and SuperScript™ II Reverse Transcriptase from Invitrogen following the manufacturer's manual. qRT‐PCR analysis of cDNA was carried out in triplicate using Brilliant III Ultra‐Fast SYBR® Green QPCR Master Mix (Agilent Technologies) on a Stratagene Mx3005P (Agilent Technologies). The reactions were performed in 20‐μl final volume with 10 μl of 2x SYBR Green QPCR master mix, 0.5 μl of each upstream and downstream primers, 0.3 μl of reference dye, 2 μl of cDNA template (10 ng), and 6.7 μl of nuclease‐free PCR‐grade water. The thermal cycling conditions were 95°C, 10 min followed by 40 cycles of 95°C for 20 sec, and 60°C for 22 sec, then 1 cycle of 95°C for 1 min, 55°C for 30 min, and 95°C for 30 sec. The gene copy numbers were measured relative to that of a housekeeping gene (*ALG9*). Oligos used for qRT‐PCR are listed in Table S2.The fold change in gene expression of *ZWF1* was determined by relative quantification, and the calculations were made using double delta method (ΔΔCt), where ΔΔCt = (ΔCtE − ΔCtC).

### 2.10 Growth test in 96‐well microtiter plates

Precultures were prepared by inoculating a single colony in 0.5 ml defined mineral medium (pH 6.0) in 96‐deep well plate (Enzyscreen). The plate was incubated at 30°C with 250 rpm agitation at 5‐cm orbit cast overnight. Five microliter of the overnight cultures were inoculated into 150 μl of fresh medium in a new 96‐well flat bottom plate (Greiner). The plate was sealed with Breathe‐Easy® sealing membrane (Sigma‐Aldrich) and incubated at 30°C with shaking in the BioTek ELx808 microplate reader (BioTek), and the absorbance was measured at 630 nm wavelength every 10 min for 42 hr. Experiments were done in five biological replicates, and the maximum specific growth rates were calculated in the exponential growth phase.

## 3 RESULTS AND DISCUSSION

### 3.1 Validation of the method for simultaneous expression of multiple genes at different levels

We aimed to develop a method that would allow simultaneous perturbation of multiple genetic targets. For this, we decided to combine the advantages of CRISPR/Cas9, *in vivo* recombination, USER assembly, and RNAi. CRISPR/Cas9 system was used to introduce a double‐strand break into a specific genome region, then overexpression and RNAi knock‐down constructs were assembled and integrated into this genome region by homologous recombination. To enable the assembly, we designed 60 bp synthetic homologous recombination (SHR) sequences like following. We have used the UPTAG and DNTAG sequences from yeast knockout libraries to design the SHR sequences. We recombined 20 bp‐UPTAG and DNTAG sequences from yeast knock‐out library (Giaever and Nislow 2014) to obtain final sequences of 60 bp. These sequences were BLASTed against S. cerevisiae genome to select the sequences with low homology that were used as overhang sequences for assembly.

**Figure 1:**
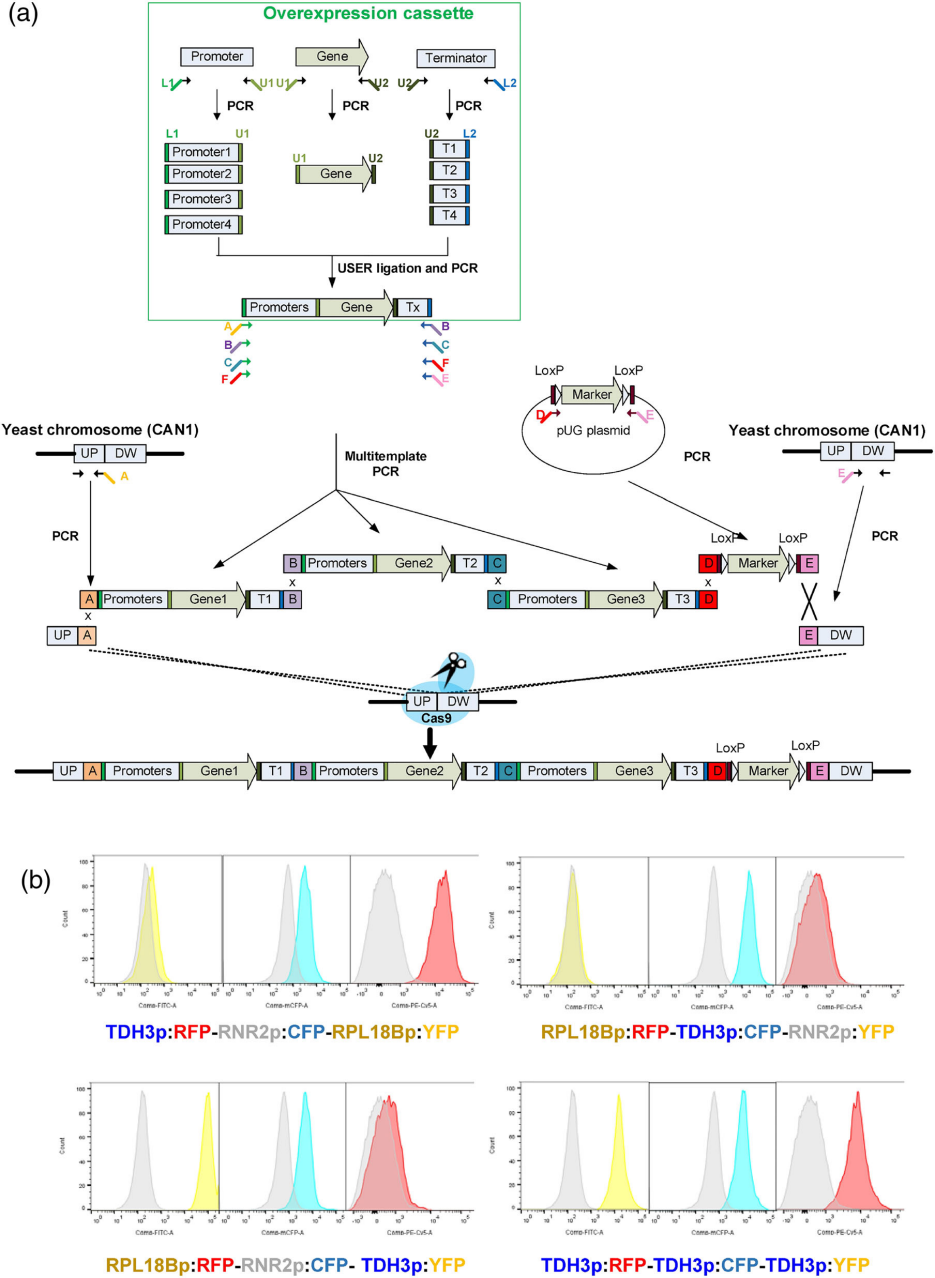
Method for expression of multiple genes. (a) Overview of the CRISPR/Cas9‐RNA interference workflow for expressing multiple genes. First, expression constructs are assembled using USER cloning‐ligation‐PCR. The promoter and terminator are chosen to obtain the desired gene expression level. In the next step, the expression constructs are transformed into Cas9‐expressing yeast strain, along with upstream and downstream repair fragments and a selective marker. (b) Fluorescent cytometry analysis of four Saccharomyces cerevisiae strains, where genes encoding for red (RFP), cyan (CFP), and yellow (YFP) fluorescent proteins were expressed under control of promoters with different strengths [Colour figure can be viewed at wileyonlinelibrary.com]

The gene BioBricks included standard 6–8 bp USER overhangs for easy assembly with promoters and terminators (Figure 1a). The promoter biobricks included standard 18 bp overhang (L1) at the 5′‐end and 6–8 bp USER overhang at 3′‐end. Similarly, the terminator biobricks also included standard 6–8 bp USER overhang and 18 bp overhang (L2) at 5′‐ and 3′‐end, respectively. The standard overhangs L1 and L2 were combined with the SHR sequences and used as primers for amplification of the assembled expression cassettes. This design allows reusing a standard set of primers for amplification of different genes, so the genes can be combined with different promoter/terminator pair. There is also a standard set of primers for amplification of expression cassettes that can be combined in the desired order. We used a range of promoters of different strengths (Table S2) and terminators. In order to validate the method for expressing multiple genes, we introduced three fluorescent protein‐coding genes (CFP, YFP, and RFP) under control of promoters of varying strength. A S. cerevisiae strain CEN.PK2‐1C (Mata *ura3 his3 leu2 trp1*) expressing Cas9p (*TRP1* selection) was transformed with gRNA (*LEU2* selection) targeting CAN1 site and with three overexpression cassettes, marker cassette (*KlURA3*), and up‐ and down‐fragments of *CAN1*. The CAN1 site was chosen because it allows easy validation of correct integration on selective plates, but as such, any site can be used. For example, intergenic sites reported as EasyClone sites can be used (Jessop‐Fabre et al., 2016). The selection marker can be omitted as well if desired; this will, however, lead to a slightly higher number of nonedited clones. Transformants were selected on drop‐out plates without tryptophan, leucine, and uracil. The correct integration into the *CAN1* site was investigated by replicating the colonies on SC‐arg + canavanine plates, where only strains with disrupted *CAN1* gene can survive. More than 95% of the colonies could grow on SC‐arg + can.

Furthermore, multiplex PCR was performed to verify the correct assembly, at least 70% of the tested strains were correct according to PCR. The fluorescence levels were evaluated by fluorescent cytometry. The four designed strains expressed all three RFP, CFP, and YFP proteins at the levels that corresponded to promoter strength (*TDH3p* > *RPL18Bp* > *RNR2p*; Figure 1b).

In the past few years, several CRISPR/Cas9 mediated multiplex genome engineering approaches were demonstrated. Mans et al. (2015) explored the potential of CRISPR/Cas9 to combine gene deletion with the simultaneous *in vivo* assembly and chromosomal integration of multiple DNA fragments. A strain carrying a double *ACS1* and *ACS2* deletion combined with six gene cassettes expressing the *Enterococcus faecalis* pyruvate dehydrogenase (PDH) complex (*aceF*, *lplA2*, *lplA*, *pdhB*, *lpd*, and *pdhA*) was constructed in a single transformation with 100% efficiency. In another study, Jakočiu̅nas et al. (2015) developed CasEMBLR, a tool for highly efficient and marker‐free assembly and integration of multiple DNA components into genomic loci. One step assembly and integration of the carotenoid pathway (*CrtYB*, *CrtI*, and *CrtE*) from 15 DNA parts (upstream homology arm, promoter, CDS, terminator, and downstream homology arm) into three targeted loci (*ADE2*, *HIS3*, and *URA3*) was demonstrated with the 31% efficiency. Furthermore, CasEMBLR was also used to assemble and integrate the five‐part assembly of the ARO4* and ARO7* expression cassettes into genomic *PDC5* and *ARO10* loci with an average efficiency of 58%. Our method is not essentially different from the previous studies but provides an advantage of standardized design of primer overhangs and consequently facilitates combinatorial assembly of genes and promoters/terminators.

### 3.2 Validation of the method for downregulation of gene expression using RNAi

**Figure 2:**
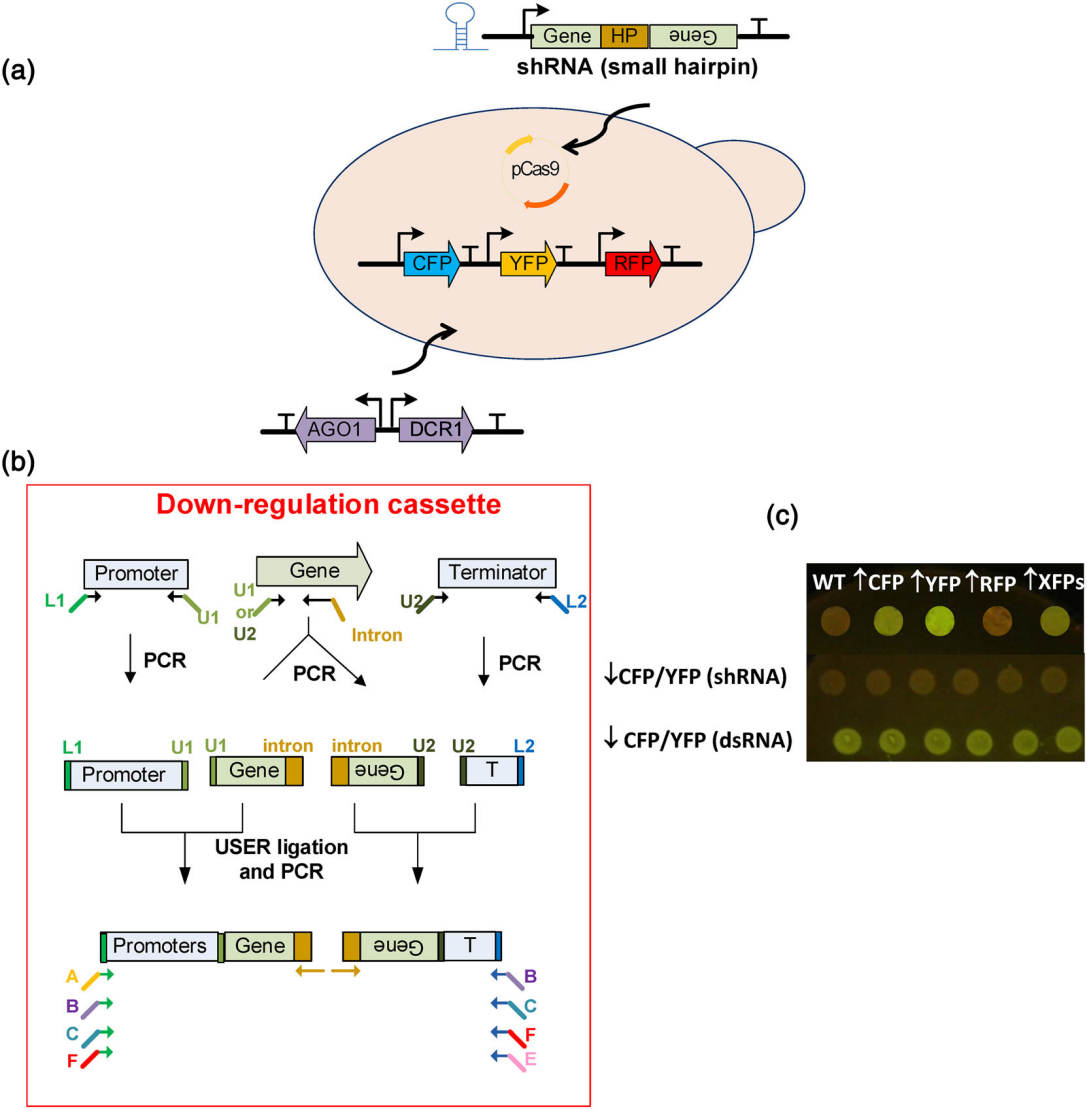
Method for downregulation of target genes. (a) Two heterologous genes AGO1 and DCR1 from Naumovozyma castellii were overexpressed in a yeast strain already expressing Cas9 and CFP‐YFP‐RFP genes. (b) Schematic illustration of USER assembly of the downregulation cassette. (c) Fluorescence images of yeast colonies expressing either individual fluorescent proteins, three fluorescent proteins (XFP), or expressing XFPs, and a downregulation construct for CFP/YFP [Colour figure can be viewed at wileyonlinelibrary.com]

RNAi machinery is present in multiple eukaryotes, including some yeast species, such as *Naumovozyma castellii* (Crook et al., 2013; Drinnenberg et al., 2009; Suk et al., 2011). Although S. cerevisiae does not harbor an active RNAi pathway, this pathway can be restored by introducing Argonaute (*AGO1*) and Dicer (*DCR1*) genes from *Naumovozyma castellii* into the genome of S. cerevisiae. In this study, we sought to reconstitute the RNAi machinery in S. cerevisiae to allow controlled downregulation of multiple target genes. We first implemented *AGO1* and *DCR1* from *Naumovozyma castellii* into S. cerevisiae through genomic integration and further expressed *Cas9* in the engineered strain from a CEN/ARS plasmid (Figure 2a). For the proof‐of‐concept, we chose to use fluorescent proteins as a reporter system. Three fluorescent protein‐encoding genes under control of strong constitutive promoters were integrated into the genome of the yeast strain with *AGO1/DCR1/Cas9* to obtain strain ST3135 for testing RNAi.

To test the capability of RNA silencing in S. cerevisiae, we evaluated two different approaches, shRNAs and dsRNAs, to silence CFP and YFP. Due to the nucleotide sequence homology between *CFP* and *YFP*, we designed shRNA and dsRNA constructs to target both genes simultaneously. The shRNA constructs contained inverted repeats of 250‐bp parts of the target gene with a hairpin in between (Figure 2b). The dsRNA construct contained the target gene flanked by convergent promoters to generate a dsRNA transcript. Both silencing constructs were under the control of strong constitutive promoters. A significant knockdown of CFP/YFP expression was observed with shRNA construct of CFP/YFP, but not with dsRNA construct (Figure 2c). These results confirmed that the RNAi mechanism is functional in S. cerevisiae, and the highest level of RNA silencing was obtained from hairpin constructs, which was in line with the previous reports. Drinnenberg et al. (2009) restored the functional RNAi system in S. cerevisiae by heterologous expression of *AGO1* and *DCR1*. The two constructs shRNA and dsDNA were designed to silence a green fluorescent protein (GFP) reporter, and shRNA has been reported to be the stronger silencing construct compared with dsRNA, both at RNA and fluorescence levels. Furthermore, Crook et al. (2013) studied several design principles for the construction of hairpin RNA expression cassettes and reported that the RNAi efficiency was improved with increasing hairpin length and demonstrated the effectiveness of RNAi by testing several genetic targets for improvement of itaconic acid production in three strains of S. cerevisiae.

In our study, the hairpin length of approximately 250 bp was used. It should also be noted that *in vivo* assembly of sense and antisense fragments provides a more straightforward approach to introduce shRNA compared with the cloning of inverted repeats via restriction‐ligation cloning in E. coli as in Yoshimatsu and Nagawa (1989).

### 3.3 Engineering CCM production through multiplex engineering

**Figure 3:**
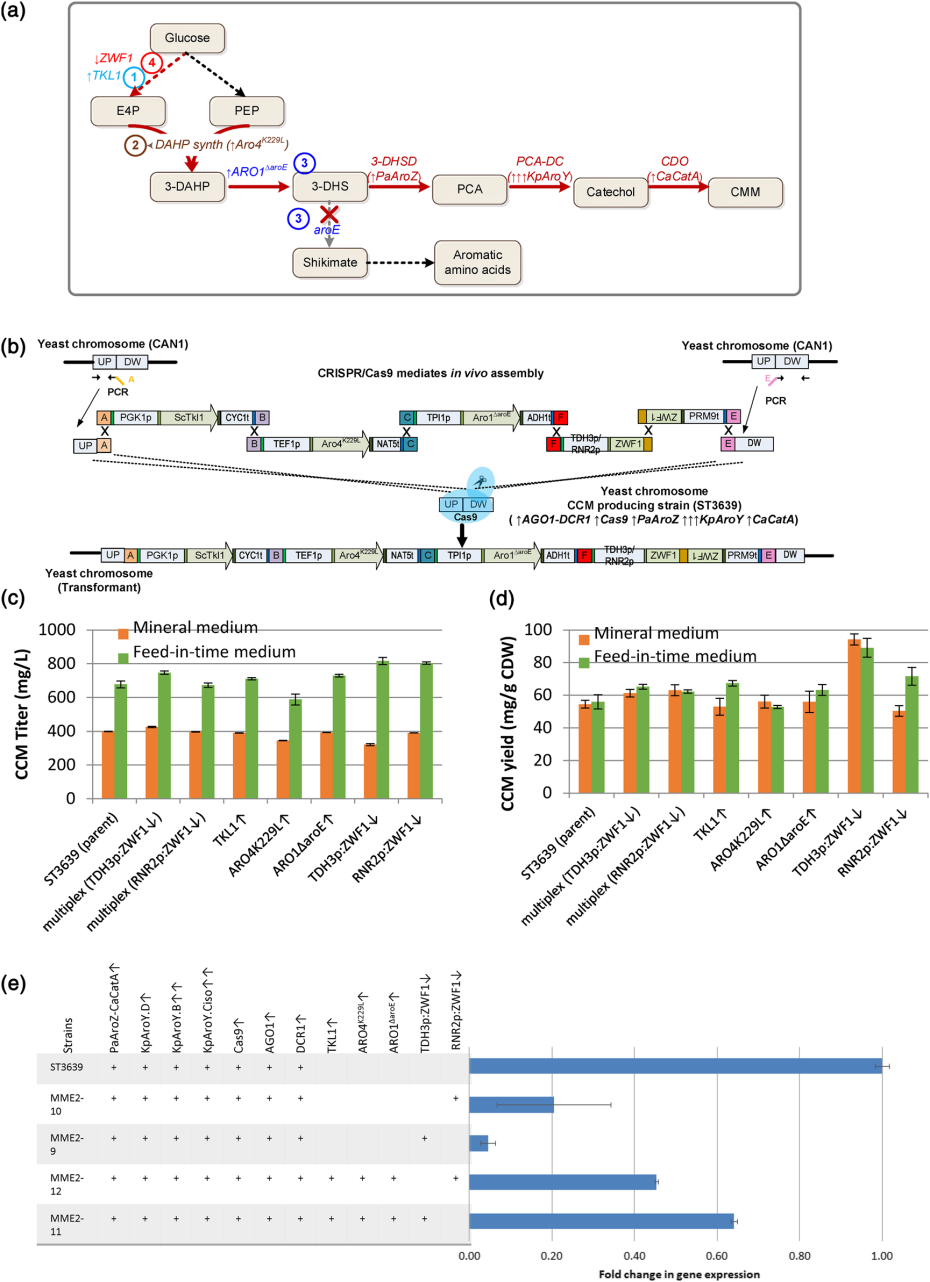
Application of CRISPR/Cas9‐RNA interference method for engineering cis,cis‐muconic acid production in Saccharomyces cerevisiae. (a) Muconic biosynthesis pathway in yeast. (b) Schematic illustration of the seven‐part assembly of the three overexpression cassettes for TKL1, ARO4 ^K229L^, ARO1 ^ΔaroE^, one downregulation cassette of ZWF1, and homologous recombination with chromosomal target site CAN1. (c, d) Average cis,cis‐muconic acid titers and yields, respectively, in the parent strain ST3639 and engineered strains with either expression of TKL1, ARO4 ^K229L^, ARO1 ^ΔaroE^, downregulation of ZWF1 or multiplex expression of all combinations. Cultivations were performed in biological triplicates, and error bars represent the standard deviation of the average (n = 3). (e) qRT‐PCR analyses. Fold change in gene expression of engineered strains compared with the parent strain ST3639. ↑ indicates that a gene was expressed in a copy, ↑↑ indicates that a gene was expressed in several copies, ↓ indicates downregulation of ZWF1 under control of either TDH3p or RNR2p promoters. Error bars represent the standard deviation of duplicates [Colour figure can be viewed at wileyonlinelibrary.com]

In the previous study, we have constructed a S. cerevisiae CCM producing strain ST3058 (Skjoedt et al., 2016). ST3058 expresses a three‐step heterologous pathway consisting of a gene encoding dehydroshikimate dehydratase (3‐DHS) from *Podosporaanserine* (*PaAroZ*), the genes encoding three different subunits of PCA‐DC from *Klebsiella pneumonia* (*KpAroY.B, KpAroY.Ciso*, *KpAroY.D*), and the gene encoding catechol 1,2‐dioxygenase (CDO) from Candida albicans (*CaCatA*; Figure 3a). It has been reported that PCA‐DC was a rate‐limiting step for the CCM flux (Curran, Leavitt, Karim, & Alper, 2013; Weber et al., 2012). For this reason, we integrated *KpAroY.B* and *KpAroY.Ciso* genes in multiple copies into long 113 terminal repeats (LTRs) of retrotransposon of the TY4 family (Maury et al., 2016). As the transformants were expected to have different copy numbers of the expression vector, we screened 12 randomly selected clones to test for CCM production. The best isolate of ST3058 produced 400 mg L^−1^ CCM in defined mineral medium and was chosen for evaluating the CRISPR/Cas9‐RNAi method. We implemented *Cas9*, *AGO1,* and *DCR1* into the best isolate of ST3058, resulting in strain ST3639 that was suitable for testing our method.

For the test, we designed to vary the expression of four native genes that could influence the CCM flux: *TKL1* encoding transketolase, *ARO4* ^*K229L*^ encoding tyrosine‐feedback‐resistant allele of phospho‐2‐dehydro‐3‐deoxyheptonate aldolase, *ARO1* ^*∆aroE*^ encoding a pentafunctional AROM protein *ARO1* without the dehydrogenase domain *AROE*, and *ZWF1* encoding glucose‐6‐phosphate dehydrogenase (Figure 3b). We generated seven strain variants that carried overexpressions of either *TKL1*, *ARO4* ^*K229L*^, *ARO1* ^*ΔaroE*^ or downregulations of *ZWF1*, or a combination of overexpressions and downregulation. For verification of correct assembly and integration, multiplex PCR of a minimum of 12 colonies per transformation was used. On the basis of genotyping, we obtained engineering efficiencies of at least 85% for *in vivo* assembly and integration of three DNA fragments (upstream homology arm, single expression cassette, and downstream homology arm), whereas 55% efficiency was obtained for combinatorial multiplex genome integration of seven DNA fragments. Several strain variants, that is, strains with downregulation of *ZWF1*, had higher CCM titer and specific yield than the parental strain ST3639. The improvement in CCM production in the engineered strains was more pronounced on feed‐in‐time medium simulating carbon‐limited fed‐batch conditions than in a standard batch medium. Overexpression of either *TKL1* or *ARO1* ^*ΔaroE*^ and downregulation of *ZWF1* with either strong or weak promoter (*TDH3p* and *RNR2p*) improved the titer by 5–21%, and the specific yield by 11–60% when the strains were grown on feed‐in‐time medium (Figure 3c,d). Contrary, overexpression of the *ARO4* ^*K229L*^ gene had no positive effect on CCM titer and yield. We also measured the μ_max_ of the four strains with *ZWF1* downregulation. No significant difference was observed in the *ZWF1* downregulation strains in comparison with the reference strain (Figure S1). However, *ZWF1* downregulation did result in a reduction of the biomass yield in comparison with the reference strain. This observation might explain the significant improvement in specific CCM yield in strains with downregulation of *ZWF1* (*TDH3*p). The downregulation of *ZWF1* gene was investigated by qRT‐PCR (Figure 3e). In the strain, where the only implemented modification was *ZWF1* downregulation, the expression level decreased by 80% or 95% when weak and strong promoters were driving shRNA expression, respectively. In the strain, where additional three genes were overexpressed, the downregulation of *ZWF1* was at 35% or 55%, again depending on the promoter for shRNA. The positive effects of *TKL1* overexpression and *ZWF1* downregulation on CCM production are in agreement with a previous report, where *ZWF1* was though deleted rather than downregulated (Curran et al., 2013; Weber et al., 2012). Both genes are involved in the pentose phosphate pathway, and the modification of their expression possibly improved the supply of the aromatic amino acids precursor—erythrose 4‐phosphate. The positive effects of these modifications need to be further confirmed in fed‐batch fermentations in controlled bioreactors.

In the past few years, there has been a growing interest in applying CRISPR methods for combinatorial metabolic engineering. Vanegas, Lehka, and Mortensen (2017) developed a Cas9/dCas9 based system, SWITCH, which allows S. cerevisiae strains to alternate between a genetic engineering state and a pathway control state. The Cas9 system was first used in the genetic engineering state to implement the five genes necessary for naringenin production into the chromosome. Next, the cells were switched to pathway control state by replacing the Cas9 expression cassette with dCas9 expression cassette. At this state, the naringenin production was further optimized by dCas9‐mediated downregulation of an essential gene *TSC13* to prevent for formation of a by‐product. However, the SWITCH approach only allows the cells to be in either a genetic engineering or a pathway control state at a time.

In another study, Lian et al. (2017) developed a trifunctional CRISPR system that combines one nuclease‐deficient CRISPR protein fused with an activation domain for transcriptional activation (CRISPRa), a second nuclease‐deficient CRISPR protein fused with a repression domain for transcriptional interference (CRISPRi), and a third catalytically active CRISPR protein for gene deletion (CRISPRd) in the same cells. Lian et al. characterized several CRISPR orthologs in S. cerevisiae and further optimized for transcriptional regulation by engineering the corresponding effector domains. The optimal design of the trifunctional CRISPR system was using nuclease‐deficient Cpf1 from *Lachnospiraceae bacterium* (dLbCpf1‐VP) for CRISPRa, nuclease‐deficient Cas9 from Streptococcus pyogenes (dSpCas9‐RD1153) for CRISPRi, and Cas9 from Staphylococcus aureus (SaCas9) for CRISPRd. As a proof‐of‐ concept, the trifunctional CRISPR system was used to increase β‐carotene production via simultaneous upregulation of *HMG1*, downregulation of *ERG9*, and deletion of *ROX1*. Furthermore, 2.5‐fold improvement in the display of an endoglucanase on the yeast surface was obtained by combinatorial optimization of several metabolic targets. At this point, the selection of efficient gRNA for CRISPRi remains a challenge and multiple variants need to be tested. This increases the number of strains that need to be constructed for testing downregulation targets or combinations of downregulation targets with overexpression targets.

During this work, a study was published by Si et al. (2017) that reported a combination of RNAi and CRISPR/Cas9 for constructing S. cerevisiae strains with overexpressions and downregulations. The authors used δ‐regions for integration of the constructs, and hence the obtained strains are not defined as in our method but have varying numbers of different expression/downregulation cassettes integrated. Si et al. applied dsDNA constructs for RNAi, whereas in our study, shDNA were shown to be more effective for downregulating gene expression.

Our method combines the advantages of RNAi for precise downregulation, of CRISPR/Cas9 for efficient genomic integration and of yeast homologous recombination for the multiple fragment assembly. The method is convenient for testing defined combinations of multiple upregulation and downregulation targets for metabolic engineering. The method can facilitate the strain development efforts by increasing the throughput and decreasing the cost of strain construction. In the future, it can be further applied for generating combinatorial libraries of strain variants by using mixes of BioBricks rather than specific BioBricks. The library approach is particularly attractive if a high‐throughput method for screening the strain libraries is available, as is the case with muconic acid, where a biosensor has been reported (Skjoedt et al., 2016).

## CONFLICT OF INTEREST

The authors declare no conflict of interests.

## Supporting information


**Figure S1.** The maximum specific growth rate (μ_max_) of the recombinant strains grown in defined mineral medium in 96‐well plate. Data bars show the mean and error bars show the standard deviations calculated from five biological replicates.
**Table S1.** List of strains
**Table S2.** List of primers
USER‐specific overhangs are marked in bold, standardized overhangs L1 and L2 are marked in blue and green texts, respectively. SHR overhang are underlined.
**Table S3.** Summary of overhangs used for amplification of biobrick parts for in vivo assembly. USER‐specific overhangs are marked in bold, standardized overhangs L1 and L2 are marked in blue and green texts, respectively. Intron sequences are in orange texts. AAAACA – Kozak sequence, **ATG**– start codon, TCA – stop codon, (N)_n_ – gene (promoter)‐specific sequence_._
**Table S4.** List of BioBricks
**Table S5.** List of plasmids
