# Supplementary material for: CRISPR/Cas9‐RNA interference system for combinatorial metabolic engineering of Saccharomyces cerevisiae
Source: Yeast. 2019 Jun 13;36(5):237–47. doi: 10.1002/yea.3390 (PMC6619288; doi:10.1002/yea.3390)
Supplement: Supplementary file 1 — Figure S1. The maximum specific growth rate (μmax) of the recombinant strains grown in defined mineral medium in 96‐well plate. Data bars show the mean and error bars show the standard deviations calculated from five biological replicates. Table S1. List of strains Table S2. List of primers USER‐specific overhangs are marked in bold, standardized overhangs L1 and L2 are marked in blue and green texts, respectively. SHR overhang are underlined. Table S3. Summary of overhangs used for amplification of biobrick parts for in vivo assembly. USER‐specific overhangs are marked in bold, standardized overhangs L1 and L2 are marked in blue and green texts, respectively. Intron sequences are in orange texts. AAAACA – Kozak sequence, ATG– start codon, TCA – stop codon, (N)n – gene (promoter)‐specific sequence. Table S4. List of BioBricks Table S5. List of plasmids [file YEA-36-237-s001.docx]

**Supplementary Figures**

**Figure S1.**The maximum specific growth rate (μ_max_) of the recombinant strains grown in defined mineral medium in 96-well plate. Data bars show the mean and error bars show the standard deviations calculated from five biological replicates.

**Supplementary Tables**

**Supplementary Table S1. List of strains**

| **Name** | **Description** | **Parent strain** | **BioBricks and Plasmids** | **Reference** |
| --- | --- | --- | --- | --- |
|  |  |  |  |  |
| CEN.PK113-7D | MAT*aMAL2-8^c^ SUC2* |  |  | Peter Kötter |
| CEN.PK102-5B | MAT*a ura3-52 his3∆1 leu2-3/112 MAL2-8^c^ SUC2* |  |  | Peter Kötter |
| CEN.PK 2-1C | MAT*a ura3-52 his3∆1 leu2-3/112 trp1-289 MAL2-8^c^ SUC2* |  |  | Peter Kötter |
| *Naumovozyma castellii* CLIB290 |  |  |  | INRA |
| **TURBO-overexpression** | | | | |
| ST2148 | MAT*a ura3-52 his3∆1 leu2-3/112 trp1-289 MAL2-8^c^ SUC2* pARS-*TEF1*p-*Cas9*-*CYC1*t | CEN.PK 2-1C | pCfB1767 | (Ronda *et al.* 2015)) |
| MME1-1 | *TDH3p*-*RFP*-*ADH1t*-*PRL18Bp*-*CFP*-*CYC1t*-*RNR2p*-*YFPopt*-*NAT5t-loxP-KlURA3-loxP*::*can1* | ST2148 | BB0629, BB0641, BB0645, BB0649, BB0627, BB0630, pCfB1791 | This study |
| MME1-2 | *TDH3p*-*RFP*-*ADH1t*-*RNR2p*-*CFP*-*CYC1t*-*PRL18Bp*-*YFPopt*-*NAT5t-loxP-KlURA3-loxP*::*can1* | ST2148 | BB0629, BB0641, BB0646, BB0648, BB0627, BB0630, pCfB1791 | This study |
| MME1-3 | *PRL18Bp*-*RFP*-*ADH1t*-TDH3*p*-*CFP*-*CYC1t*-*RNR2p*-*YFPopt*-*NAT5t-loxP-KlURA3-loxP*::*can1* | ST2148 | BB0629, BB0642, BB0644, BB0649, BB0627, BB0630, pCfB1791 | This study |
| MME1-4 | *PRL18Bp*-*RFP*-*ADH1t*-*RNR2p*-*CFP*-*CYC1t*-*TDH3p*-*YFPopt*-*NAT5t-loxP-KlURA3-loxP*::*can1* | ST2148 | BB0629, BB0642, BB0646, BB0647, BB0627, BB0630, pCfB1791 | This study |
| MME1-5 | *RNR2p*-*RFP*-*ADH1t*-*TDH3p*-*CFP*-*CYC1t*-*PRL18Bp*-*YFPopt*-*NAT5t-loxP-KlURA3-loxP*::*can1* | ST2148 | BB0629, BB0643, BB0644, BB0648, BB0627, BB0630, pCfB1791 | This study |
| MME1-6 | *RNR2p*-*RFP*-*ADH1t*-*PRL18Bp*-*CFP*-*CYC1t*-*TDH3p*-*YFPopt*-*NAT5t-loxP-KlURA3-loxP*::*can1* | ST2148 | BB0629, BB0643, BB0645, BB0647, BB0627, BB0630, pCfB1791 | This study |
| MME1-7 | *TDH3p*-*RFP*-*ADH1t*-*TDH3p*-*CFP*-*CYC1t*-*TDH3p*-*YFPopt*-*NAT5t-loxP-KlURA3-loxP*::*can1* | ST2148 | BB0629, BB0641, BB0644, BB0647, BB0627, BB0630, pCfB1791 | This study |
| MME1-8 | *PRL18Bp*-*RFP*-*ADH1t*-*TDH3p*-*CFP*-*CYC1t*-*RNR2p*-*YFPopt*-*NAT5t-loxP-KlURA3-loxP*::*can1* | ST2148 | BB0629, BB0642, BB0644, BB0649, BB0627, BB0630, pCfB1791 | This study |
| MME1-9 (negative control strain) | *TDH3p*-*CFP*-*CYC1t*-*RNR2p*-*YFPopt*-*NAT5t-loxP-KlURA3-loxP*::*can1* | ST2148 | BB0629, BB0644, BB0649, BB0627, BB0630, pCfB1791 | This study |
| MME1-10 (negative control strain) | *loxP-KlURA3-loxP*::*can1* | ST2148 | BB0627, pCfB1791 | This study |
| MME1-11 | pX-2-*KlURA3*, pX-3-*KlLEU2*, pX-4-*SpHis5* | CEN.PK102-5B | pCfB255, pCfB257, pCfB258 | This study |
| MME1-12 | pX-2-*KlURA3-CFP* pX-3-*KlLEU2-RFP* pX-4-*SpHis5-YFP* | CEN.PK102-5B | pCfB393, pCfB394, pCfB395 | This study |
| ST6253 | pX-2-*KlURA3-CFP* pX-3-*KlLEU2* pX-4-*SpHis5* | CEN.PK102-5B | pCfB393, pCfB257, pCfB258 | This study |
| ST6254 | pX-2-*KlURA3* pX-3-*KlLEU2-RFP* pX-4-*SpHis5* | CEN.PK102-5B | pCfB255, pCfB258, pCfB394 | This study |
| ST6255 | pX-2-*KlURA3* pX-3-*KlLEU2* pX-4-*SpHis5-YFP* | CEN.PK102-5B | pCfB255, pCfB257, pCfB395 | This study |
| **TURBO- RNAi** | | | | |
| ST3098 | pX-3-*KlURA3-AGO1*-*DCR1* | CEN.PK 2-1C | pCfB284 | This study |
| ST3133 | pX-3-*KlURA3-AGO1*-*DCR1* pX-4-*SpHis5*-*YFP* pXI-1- KlLEU2*-*RFP*-CFP* | ST3098 | pCfB395, pCfB2586 | This study |
| ST3134 | pX-3-*KlURA3-AGO1*-*DCR1* pX-4-*YFP* pXI-1-*RFP*-*CFP* | ST3133 | pCfB61 | This study |
| ST3135 | pX-3-*KlURA3*-*AGO1*-*DCR1* pX-4-*YFP* pXI-1-*RFP*-*CFP* pRS414-TRP1-Cas9 | ST3134 | pCfB1767 | This study |
| ↓CFP/YFP (shRNA) | pX-3-*KlURA3*-*AGO1*-*DCR1* pX-4-*YFP* pXI-1-*RFP*-*CFP* p414-*TRP1*-*Cas9* ↓*CFP/YFP-SpHIS5*::*can1* | ST3135 | pCfB1791, BB0629, A-YFP/CFP-sense, YFP/CFP-antisense-D, BB0866, BB0630 | This study |
| ↓CFP/YFP (dsRNA) | pX-3-*KlURA3*-*AGO1*-*DCR1* pX-4-*YFP* pXI-1-*RFP-CFP* p414-*TRP1*-*Cas9* ↓CFP/YFP*-SpHIS5*::*can1* | ST3135 | pCfB1791, BB0629, A-CFP/YFP_convergent-D, BB0866, BB0630 | This study |
| A-SpHIS5-E (negative control) | pX-3-*KlURA3*-*AGO1*-*DCR1* pX-4-*YFP* pXI-1-*RFP*-*CFP* pRS414-TRP1-Cas9 *SpHIS5::can1* | ST3135 | pCfB1791, BB0629, BB1019, BB0630 | This study |
| **TURBO-CCM** | | | | |
| ST-3054 | pXI-1-*KlLEU2*-*PaAroZ*-C*aCatA* pX-4-*SpHIS5*-*KpAroY.D* | CEN.PK102-5B | pCfB1239, pCfB2695 | Skjoedt et al. 2016 |
| ST-3058 | pXI-1-*KlLEU2*-*PaAroZ*-*CaCatA* pX-4-*SpHiS5*-*KpAroY.D* pTY4-*KlURA3*-*KpAroY.B*-*KpAroY.Ciso* | ST-3054 | pCfB1241 | Skjoedt et al. 2016 |
| ST-3636 | pXI-1-*KlLEU2*-*PaAroZ*-*CaCatA* pX-4-*SpHiS5*-*KpAroY.D* pTY4-*KlURA3*-*KpAroY.B*-*KpAroY.Ciso* pRS414-*KanMX-Cas9* | ST-3058 | pCfB2312 | This study |
| ST-3639 | pXI-1-*KlLEU2*-*PaAroZ*-*CaCatA* pX-4-*SpHIS5*-*KpAroY.D* pTY4-*KlURA3*-*KpAroY.B*-*KpAroY.Ciso* pRS414-*KanMX*-*Cas9* pX-3-*AGO1*-*DCR1* | ST-3636 | pCfB3041, pCfB3100 | This study |
| MME2-6 (TKL1↑) | pXI-1-*KlLEU2*-*PaAroZ*-*CaCatA* pX-4-*SpHIS5*-*KpAroY.D* pTY4*-KlURA3*-*KpAroY.B-KpAroY.Cis*o pRS414*-KanM*X*-Cas*9pX-3-*AGO1*-*DCR↑1 TKL1↑:can1* | ST-3639 | BB0629, A-TKL1-E, BB0630 | This study |
| MME2-7 (ARO4^K229L^↑) | pXI-1-*KlLEU2*-*PaAroZ*-*CaCatA* pX-4-*SpHIS5*-*KpAroY.D* pTY4*-KlURA3*-*KpAroY.B-KpAroY.Cis*o pRS414-KanMX-Cas9pX-3-*AGO1*-*DCR1 ↑ARO4^K229L^::can1* | ST-3639 | BB0629, A-ARO4^K229L^-E, BB0630 | This study |
| MME2-8 (ARO1^∆aroE^↑) | pXI-1-*KlLEU2*-*PaAroZ*-*CaCatA* pX-4-*SpHIS5*-*KpAroY.D* pTY4*-KlURA3*-*KpAroY.B-KpAroY.Cis*o pRS414-KanMX-Cas9pX-3-*AGO1*-*DCR1 ↑ARO1^∆aroE^ ::can1* | ST-3639 | BB0629, A-ARO1∆^aroE^-E, BB0630 | This study |
| MME2-9 (TDH3p:ZWF1↓) | pXI-1-*KlLEU2*-*PaAroZ*-*CaCatA* pX-4-*SpHIS5*-*KpAroY.D* pTY4-*KlURA3-KpAroY.B-KpAroY.Cis*o pRS414*-KanM*X*-Cas*9pX-3-*AGO1*-*DCR1 TDH3p:ZWF1↓::can1* | ST-3639 | BB0629, A-TDH3p-ZWF1_sense, ZWF-antisense-E, BB0630, pCfB2310 | This study |
| MME2-10 (RNR2p:ZWF1↓) | pXI-1-*KlLEU2*-*PaAroZ*-*CaCatA* pX-4-*SpHIS5*-*KpAroY.D* pTY4*-KlURA3*-*KpAroY.B-KpAroY.Cis*o pRS414*-KanM*X*-Cas*9pX-3*-AGO1-DCR1 RNR2p:ZWF1↓::can1* | ST-3639 | BB0629, A-RNR2p-ZWF1_sense, ZWF-antisense-E, BB0630, pCfB2310 | This study |
| MME2-11 (multiplex TDH3p:ZWF1↓) | pXI-1*-KlLEU2*-*PaAroZ*-*CaCatA* pX-4-*SpHIS5*-*KpAroY.D* pTY4-*KlURA3*-*KpAroY.B-KpAroY.Cis*o pRS414*-KanM*X*-Cas*9pX-3*-AGO1-DCR1 ↑TKL1 ↑ARO4^K229L^ ↑ARO1^∆aroE^ ↓TDH3p:ZWF1::*can1 | ST-3639 | BB0629, A-TKL1-B, B-ARO4^K229L^-C, C-ARO1^∆aroE^-F, F-TDH3p-ZWF1_sense, ZWF-antisense-E, BB0630, pCfB2310 | This study |
| MME2-12 (multiplex RNR2p:ZWF1↓) | pXI-1-*KlLEU2*-*PaAroZ*-*CaCatA* pX-4-*SpHiS5*-*KpAroY.D* pTY4*-KlURA3-KpAroY*.B*-KpAroY.Cis*o pRS414*-KanM*X*-Cas*9pX-*3-AGO1-DCR1 ↑TKL1 ↑ARO4^K229L^ ↑ARO1^∆aroE^ ↓RNR2p:ZWF1::can1* | ST-3639 | BB0629, A-TKL1-B, B-ARO4^K229L^-, C-ARO1^∆aroE^-F, F-RNR2p-ZWF1_sense, ZWF-antisense-E, BB0630, pCfB2310 | This study |

**Supplementary Table S2. List of primers**

USER-specific overhangs are marked in bold, standardized overhangs L1 and L2 are marked in blue and green texts, respectively. SHR overhang are underlined.

| **Primer ID** | **Name** | **Sequence (5'->3')** |
| --- | --- | --- |
| PR-6 | TEF1p_rv | **CACGCGAU**GCACACACCATAGCTTC |
| PR-8 | PGK1p_rv | **ATGACAGAU**TTGTTTTATATTTGTTG |
| PR-496 | AGO1_U_fw | **AGTGCAGGU**AAAACAatgtcatccaattcggaggag |
| PR-497 | AGO1_U_rv | **CGTGCGAU**tcatatgtagtacatgatgtcag |
| PR-498 | DCR1_U_fw | **ATCTGTCAU**AAAACAatgaatagagaaaaaagcgccgatc |
| PR-499 | DCR1_U_rv | **CACGCGAU**tcacagattgttgcaatgcctc |
| PR-1388 | aro4_U1_fw | **AGTGCAGGU**AAAACAATGAGTGAATCTCCAATGTTCG |
| PR-1400 | tkl1_U2_fw | **ATCTGTCAU**AAAACAATGACTCAATTCACTGACATTG |
| PR-1401 | tkl1_U2_rv | **CACGCGAU**TCAGAAAGCTTTTTTCAAAGGAG |
| PR-1402 | tal1_U1_fw | **AGTGCAGGU**AAAACAATGTCTGAACCAGCTCAAAAG |
| PR-1403 | tal1_U1_rv | **CGTGCGAU**TCAAGCGGTAACTTTCTTTTCAATC |
| PR-3109 | YFP/CFP_F+ | **AGTGCAGGU**AAAACAATGAGTAAAGGAGAAGAACTTTTCAC |
| PR-3110 | YFP/CFP_R+ | **CGTGCGAU**TCATTTGTATAGTTCATCCATGCCATG |
| PR-3111 | RFP_F+ | **AGTGCAGGU**AAAACAATGGCCTCCTCCGAGGACGTCATC |
| PR-3112 | RFP_R+ | **CGTGCGAU**TCAGGCGCCGGTGGAGTGGCGG |
| PR-6726 | ScAro1deltaAroE_U1_fw | **AGTGCAGGU**AAAACAATGGTGCAGTTAGCCAAAG |
| PR-7540 | ASR_044_F (CAN1_UP_fw) | CTCTATCAATGAAAATTTCGAGGA |
| PR-7545 | ASR_049_R (CAN1_DW_rv) | GCTAAAACTTTGATGGAAGCG |
| PR-7566 | RFP_MME_rv | **ACGTGCGAU**TCAGGCGCCGGTGGAGTGGCGG |
| PR-7567 | CFP_MME_rv | **ACGTGCGAU**TCATTTGTATAGTTCATCCATGCCATG |
| PR-7568 | YFPopt_MME_fw | **AGTGCAGGU**AAAACAATGTCCAAGGGTGAAGAAT |
| PR-7569 | YFPopt_MME_rv | **ACGTGCGAU**TTACTTGTATAATTCGTCCATACCA |
| PR-7570 | TDH3p_fw | ATCACTCGTACAGCGGTAATAAAAAACACGCTTTTTCAGTTCG |
| PR-7571 | TDH3p_rv | **ACCTGCACU**TTTGTTTGTTTATGTGTGTTTATTCGA |
| PR-7572 | RPL18Bp_fw | ATCACTCGTACAGCGGTAGGCGTCGTTGTTAATTTCG |
| PR-7573 | RPL18Bp_rv | **ACCTGCACU**TTTGTTTTTTGTTTTCTTCTAATTGA |
| PR-7574 | RNR2p_fw | ATCACTCGTACAGCGGTACGATTTCTCATCATATCGCTATCGC |
| PR-7575 | RNR2p_rv | **ACCTGCACU**GGTAATTGGACAAATAAATACGTGTATTAAG |
| PR-7580 | ADH1t_fw | **ATCGCACGU**GCGAATTTCTTATGATTTATG |
| PR-7581 | ADH1t_rv | GGCTGAGTACAACTAGCTGAGCGACCTCATGCTATA |
| PR-7582 | CYC1t_fw | **ATCGCACGU**ATCCGCTCTAACCGAAAAG |
| PR-7583 | CYC1t_rv | GGCTGAGTACAACTAGCTTTCTCAAGCAAGGTTTTC |
| PR-7584 | NAT5t_fw | **ATCGCACGU**ATTTCTTAACAGATGGCTG |
| PR-7585 | NAT5t_rv | GGCTGAGTACAACTAGCTTCGGGACCATAAAAATTC |
| PR-8481 | PGK1p_MME_fw | ATCACTCGTACAGCGGTAGGAAGTACCTTCAAAGA |
| PR-8483 | FBA1p_rv | **ACCTGCACU**TTTGAATATGTATTACTTGGTTATGGTTA |
| PR-8493 | CFP->_U2_fw | **ATCTGTCAU**AAAACAATGAGTAAAGGAGAAGAACTTTTCAC |
| PR-8494 | CFP->_U2_rv | **CACGCGAU**TCATTTGTATAGTTCATCCATGCCATG |
| PR-10418 | NAT5t_U3_fw | **AGCTACTGAU**ATTTCTTAACAGATGGCTG |
| PR-140419 | NAT5t_L1_rv | ATCACTCGTACAGCGGTATCGGGACCATAAAAATTC |
| PR-10421 | PRM9t_L2_rv | GGCTGAGTACAACTAGCTATTTTCAACATCGTATTTTCC |
| PR-10422 | PRM9t_U2_fw | **ATCGCACGU**ACAGAAGACGGGAGACAC |
| PR-10426 | TDH3p_U2_rv | **ATCGCACGU**TTTGTTTGTTTATGTGTGTTTATTCGA |
| PR-10427 | FBA1p_U3_fw | **ATCAGTAGCU**ATAACAATACTGACAGTAC |
| PR-10435 | CFPf_U1_fw | **AGTGCAGGU**GAAGTCAAGTTTGAAGGT |
| PR-10436 | CFPf_intron_rv | GAAAAAAGTTCCAACACACCTGGTTGTCTGGTAAAAGGAC |
| PR-10437 | anti-CFPf_U2_fw | **ACGTGCGAU**GAAGTCAAGTTTGAAGGT |
| PR-10438 | anti-CFPf_intron_rv | GAAACTAATTGGTAAAGATAGTTGTCTGGTAAAAGGAC |
| PR-10439 | Intron_rad9_fw | CAGGTGTGTTGGAACTTTTTTCAAACCTTACTAAACATTGAAACTAATTGGTAAAGATA |
| PR-10440 | Intron_rad9_rv | TATCTTTACCAATTAGTTTCAATGTTTAGTAAGGTTTGAAAAAAGTTCCAACACACCTG |
| PR-10817 | Aro4K229L_newU1_rv | **ACGTGCGAU**TCATTTCTTGTTAACTTCTCTTCTTTG |
| PR-10818 | ScAro1deltaAroE_newU1_rv | **ACGTGCGAU**GATGACATTTGCGAGCTTGATGAGGAATAG |
| PR-10819 | ZWF1sense_U1_fw | **AGTGCAGGU**ATGTCCCACACCGTCTCT |
| PR-10820 | ZWF1_sense_int_rv | GAAAAAAGTTCCAACACACCTGCTCTTTACCCAAGTAATGGTCAAT |
| PR-10821 | ZWF1__antisense_U2_fw | **ACGTGCGAU**ATGTCCCACACCGTCTCT |
| PR-10822 | ZWF1_antisense_int_rv | GAAACTAATTGGTAAAGATACTCTTTACCCAAGTAATGGTCAAT |
| PR-10849 | TPI1p_L1_fw | ATCACTCGTACAGCGGTACTACTTATTCCCTTCGAGATTATATCTAG |
| PR-10850 | TPI1p_U_rv | **ACCTGCACU**TTTTAGTTTATGTATGTGTT |
| **Primers with SHR overhang for USER assemble PCR** | | |
| PR-7587 | Fusion-A_fw | ACACACTGTATAGAGGGTTGTGGATACCGCTGCACGTACTAGGATAGCGTCTCACACCATATCACTCGTACAGCGGTA |
| PR-7588 | Fusion-B_rv | ATGTCTAGGATCGTAGGTACCCGGTCTTACAGAAATGGCGCGCTCACATGACGTTACATTGGCTGAGTACAACTAGCT |
| PR-7589 | Fusion-B_fw | AATGTAACGTCATGTGAGCGCGCCATTTCTGTAAGACCGGGTACCTACGATCCTAGACATATCACTCGTACAGCGGTA |
| PR-7590 | Fusion-C_rv | CTTAGACCTGGGTAGGCTCTGAGCAGGGTTACTGACCTCTGCCCAGTCTTTCTCTATGAGGGCTGAGTACAACTAGCT |
| PR-7591 | Fusion-C_fw | CTCATAGAGAAAGACTGGGCAGAGGTCAGTAACCCTGCTCAGAGCCTACCCAGGTCTAAGATCACTCGTACAGCGGTA |
| PR-7592 | Fusion-D_rv | ATCGGGCCTGCTACATCAGTCTGACGTATTAGTCCAATGGACGACGGTGAACGGCCTAATGGCTGAGTACAACTAGCT |
| PR-11059 | Fusion-E_rv | AGTATCGGGCTCCTGGACTACTATGGCTGAGTATGTAACGTGAGACACTTTCACCCAATGGGCTGAGTACAACTAGCT |
| PR-8275 | Fusion-E_fw | CATTGGGTGAAAGTGTCTCACGTTACATACTCAGCCATAGTAGTCCAGGAGCCCGATACTATCACTCGTACAGCGGTA |
| PR-8276 | Fusion-F_rv | TCTTAGATTGTCGCTACGGCCAACTGGATATAAGTGCGCGCGACGAGATGCTCAGACTATGGCTGAGTACAACTAGCT |
| PR-8277 | Fusion-F_fw | ATAGTCTGAGCATCTCGTCGCGCGCACTTATATCCAGTTGGCCGTAGCGACAATCTAAGAATCACTCGTACAGCGGTA |
| PR-7593 | Marker-D_fw | ATTAGGCCGTTCACCGTCGTCCATTGGACTAATACGTCAGACTGATGTAGCAGGCCCGATACGCTGCAGGTCGACAAC |
| PR-7594 | Marker-E_rv | AGTATCGGGCTCCTGGACTACTATGGCTGAGTATGTAACGTGAGACACTTTCACCCAATGCTAGTGGATCTGATATCAC |
| PR-10524 | Marker_A_fw | ACACACTGTATAGAGGGTTGTGGATACCGCTGCACGTACTAGGATAGCGTCTCACACCATACGCTGCAGGTCGACAAC |
| PR-7596 | CAN1.Y_Upnew_A_rv | ATGGTGTGAGACGCTATCCTAGTACGTGCAGCGGTATCCACAACCCTCTATACAGTGTGTCTCGCCATTTACTCTCGT |
| PR-7597 | CAN1.Y_DW-E_fw | CATTGGGTGAAAGTGTCTCACGTTACATACTCAGCCATAGTAGTCCAGGAGCCCGATACTCATAGGTGATGAAGATGA |
| ***E. coli* genotyping primers** | | |
| PR-224 | ADH1_test_fw | gaaattcgcttatttagaagtgtc |
| PR-225 | CYC1_test_rv | CTCCTTCCTTTTCGGTTAGAG |
| PR-339 | TEF1_test_rv | gctcattagaaagaaagcatagc |
| PR-340 | PGK1_test_fw | TACAGATCATCAAGGAAGTAATTATC |
| **Yeast genotyping primers** | | |
| PR-897 | XII-4-out-seq_fw | GAACTGACGTCGAAGGCTCT |
| PR-903 | X-3-out-seq_fw | TGACGAATCGTTAGGCACAG |
| PR-905 | X-4-out-seq_fw | CTCACAAAGGGACGAATCCT |
| PR-907 | XI-1-out-seq_fw | CTTAATGGGTAGTGCTTGACACG |
| PR-911 | XI-3-out-seq_fw | GTGCTTGATTTGCGTCATTC |
| PR-2221 | Sc_ColoPCR_rv | GTTGACACTTCTAAATAAGCGAATTTC |
| PR-7556 | ASR_0050_F (CAN1_UP_check_fw) | AACCCTGAAATTTGCCCTATAG |
| PR-7696 | CAN1.Y_DW_check_rv | CCTTTGTACCAGAGTTCTCA |
| PR-10660 | Linker2_check_fw | AGCTAGTTGTACTCAGCC |
| **Yeast genotyping primers for multiplex PCR** | | |
| **CAN1.Y_UP+A** | | |
| PR-7556 | CAN1_UP_check_fw | AACCCTGAAATTTGCCCTATAA |
| PR-7693 | Linker 1_rv | TACCGCTGTACGAGTGAT |
| **ADH1t-B-Promoter2** | | |
| PR-7580 | TADH1_fw | ATCGCACGUGCGAATTTCTTATGATTTATG |
| PR-7693 | Linker 1_rv | TACCGCTGTACGAGTGAT |
| **CYC1t-C-Promoter3** | | |
| PR-7582 | TCYC1_fw | ATCGCACGUATCCGCTCTAACCGAAAAG |
| PR-7693 | Linker 1_rv | TACCGCTGTACGAGTGAT |
| **NATt-D-Marker** | | |
| PR-7584 | TNAT5_fw | ATCGCACGUATTTCTTAACAGATGGCTG |
| PR-7694 | Marker_check_rv | GTTGTCGACCTGCAGCGT |
| **Marker-E-CAN1.Y_Down** | | |
| PR-7695 | Marker_check_fw | GGTGATATCAGATCCACTAGC |
| PR-7696 | CAN1.Y_DW_check_rv | CCTTTGTACCAGAGTTCTCA |
| **Primers for qRT-PCR** | | |
| PR-14120 | ALG9_qPCR_fw | CCGTTGCCATGTTGTTGTATG |
| PR-14121 | ALG9_qPCR_rv | GCCAGGAAATTGTACGCTAAAC |
| PR-11778 | ZWF1_qRT_fw | TAAGCCCGCCTACGTGGATG |
| PR-11779 | ZWF1_qRT_rv | CATCATGATGGGGACGCCCT |

**Supplementary Table S3. Summary of overhangs used for amplification of biobrick parts for *in vivo* assembly.**USER-specific overhangs are marked in bold, standardized overhangs L1 and L2 are marked in blue and green texts, respectively. Intron sequences are in orange texts. *AAAACA* – Kozak sequence, **ATG**– start codon, *TCA* – stop codon, (N)_n_ – gene(promoter)-specific sequence_._

| **Description** | **Biobrick** | **Fw primer** | **Reverse primer** | **Template** |
| --- | --- | --- | --- | --- |
| **Promoter1** | Promoter1_for Gene1_EasyClone | Promoter-L1_fw  ATCACTCGTACAGCGGTANNNNNN | Promoter-U1-rv  ACCTGCAC**U**NNNNNN | gDNA of *S. cerevisiae* |
| **Terminator1** | Terminator_for Gene1_EasyClone | Terminator-U2_fw  ATCGCACG**U**NNNNNN | Terminator-L2_rv  GGCTGAGTACAACTAGCTNNNNNN | gDNA of *S. cerevisiae* |
| **Gene (over-expression)** | Gene1 | Gene-U1-fw  AGTGCAGG**U***AAAACA***ATG**NNNNNN | Gene-U2-rv  ACGTGCGA**U***TCA*NNNNN | Gene of interest |
| **Gene (down regulation; RNAi)** | Gene_sense  (~200 bp) | Gene_sense_U1-fw  AGTGCAGG**U**ATGNNNNNN | Gene_sense_int-rv  GAAAAAAGTTCCAACACACCTGNNNNNNN | Gene of interest |
|  | Gene_antisense  (reverse sequence of the Gene_sense) | Gene_antisense-U2-fw  ACGTGCGA**U**ATGNNNNN | Gene_antisense_int-rv  GAAACTAATTGGTAAAGATANNNNNNN | Gene of interest |

**Supplementary Table S4. List of BioBricks**

| **Biobrick ID** | **Description** | **Template** | **fw primer** | **rv primer** |
| --- | --- | --- | --- | --- |
| **Promoter biobricks for TURBO method** | | | | |
| BB0618 | TDH3p | gDNA *S. cerevisiae* (CEN.PK113-7D) | PR-7570 | PR-7571 |
| BB0619 | RPL18Bp | gDNA *S. cerevisiae* (CEN.PK113-7D) | PR-7572 | PR-7573 |
| BB0620 | RNR2p | gDNA *S. cerevisiae* (CEN.PK113-7D) | PR-7574 | PR-7575 |
| BB0622 | TEF1p | gDNA *S. cerevisiae* (CEN.PK113-7D) | PR-7578 | PR-7579 |
| BB0854 | FBA1p_U3-> | gDNA *S. cerevisiae* (CEN.PK113-7D) | PR-10427 | PR-8483 |
| BB0925 | TPI1p | gDNA *S. cerevisiae* (CEN.PK113-7D) | PR-10849 | PR-10850 |
| **Terminator biobricks for TURBO method** | | | | |
| BB0623 | ADH1t-> | gDNA *S. cerevisiae* (CEN.PK113-7D) | PR-7580 | PR-7581 |
| BB0624 | CYC1t-> | gDNA *S. cerevisiae* (CEN.PK113-7D) | PR-7582 | PR-7583 |
| BB0625 | NAT5t-> | gDNA *S. cerevisiae* (CEN.PK113-7D) | PR-7584 | PR-7585 |
| BB0849 | NAT5t<- | gDNA *S. cerevisiae* (CEN.PK113-7D) | PR-10418 | PR-10419 |
| BB0857 | PRM9t-> | gDNA *S. cerevisiae* (CEN.PK113-7D) | PR-10422 | PR-10421 |
| **Genes, Marker cassette, UP- and Down-fragments for TURBO method** | | | | |
| BB0615 | RFP_MME-> | pCfB394 | PR-3111 | PR-7566 |
| BB0616 | CFP_MME-> | pCfB393 | PR-3109 | PR-7567 |
| BB0617 | YFPopt_MME-> | YFPopt (gBlock® gene frgament from IDT) | PR-7568 | PR-7569 |
| BB0627 | D-KlURA3-E | p0018 (pUG72) | PR-7593 | PR-7594 |
| BB0866 | D-SpHIS5-E | pCfB258 | PR-7593 | PR-7594 |
| BB1019 | A-SpHIS5-E | pCfB258 | PR-10524 | PR-7594 |
| BB0629 | CAN1-UP-A | gDNA *S. cerevisiae* (CEN.PK113-7D) | PR-7540 | PR-7596 |
| BB0630 | CAN1-DW-E | gDNA *S. cerevisiae* (CEN.PK113-7D) | PR-7597 | PR-7545 |
| **Overexpression cassette for TURBO method** | | | | |
| BB0641 | A-TDH3p-RFP-ADH1t-B | BB0618, BB0615, BB0623 | PR-7587 | PR-7588 |
| BB0642 | A-pRPL18Bp-RFP-ADH1t-B | BB0619, BB0615, BB0623 | PR-7587 | PR-7588 |
| BB0643 | A-RNR2p-RFP-ADH1t-B | BB0620, BB0615, BB0623 | PR-7587 | PR-7588 |
| BB0644 | B-TDH3p-CFP-CYC1t-C | BB0618, BB0616, BB0624 | PR-7589 | PR-7590 |
| BB0645 | B-RPL18Bp-CFP-CYC1t-C | BB0619, BB0616, BB0624 | PR-7589 | PR-7590 |
| BB0646 | B-RNR2p-CFP-CYC1t-C | BB0620, BB0616, BB0624 | PR-7589 | PR-7590 |
| BB0647 | C-TDH3p-YFPopt-NAT5t-D | BB0618, BB0617, BB0625 | PR-7591 | PR-7592 |
| BB0648 | C-RPL18Bp-YFPopt-NAT5t-D | BB0619, BB0617, BB0625 | PR-7591 | PR-7592 |
| BB0649 | C-RNR2p-YFPopt-NAT5t-D | BB0620, BB0617, BB0625 | PR-7591 | PR-7592 |
| **Biobricks for TURBO-RNAi** | | | | |
| BB0859 | TDH3p-CYC1t | pCfB1024 | PR-10426 | PR-7583 |
| BB0860 | CFP_sense | pCfB393 | PR-10435 | PR-10436 |
| BB0861 | CFP_antisense | pCfB393 | PR-10437 | PR-10438 |
| A-YFP/CFP-sense | A-TDH3p-CFP_sense-intron1 | BB0618, BB0860 | PR-7587 | PR-10440 |
| YFP/CFP-antisense-D | intron1-CFP_antisense-ADH1t-D | BB0861, BB0623 | PR-10439 | PR-7592 |
| A-YFP/CFP_convergent-D | A-NAT5t-FBA1p-CFP-TDH3p-CYC1t-D | BB849, BB0854, BB0616, BB0859 | PR-7587 | PR-7592 |
| **Biobricks for TURBO-CCM** | | | | |
| BB0919 | ScARO4^K229L^ | pCfB775 | PR-1388 | PR-10817 |
| BB0920 | ScARO1^DaroE^ | pCfB1238 | PR-6726 | PR-10818 |
| BB0921 | ZWF1_sense | gDNA *S. cerevisiae* (CEN.PK113-7D) | PR-10819 | PR-10820 |
| BB0922 | ZWF1_antisense | gDNA *S. cerevisiae* (CEN.PK113-7D) | PR-10821 | PR-10822 |
| BB0927 | A-TDH3p-ZWF1_sense | BB0618+BB0921 | PR-7587 | PR-10440 |
| BB0928 | A-RNR2p-ZWF1_sense | BB0620+BB0921 | PR-7587 | PR-10440 |
| BB0929 | ZWF1_antisense-PRM9t-E | BB0922+BB0857 | PR-10439 | PR-11059 |
| BB0947 | PGK1p-TKL1-CYC1t | pCfB1048 | PR-8481 | PR-7583 |
| A-TDH3p-ZWF1_sense | A-TDH3p-ZWF1_sense | BB0618+BB0921 | PR-7587 | PR-10440 |
| F-RNR2p-ZWF1_sense | A-RNR2p-ZWF1_sense | BB0620+BB0921 | PR-8277 | PR-10440 |
| ZWF_antisense-RPM9t-E | ZWF1_antisense-PRM9t-E | BB0922+BB0857 | PR-10439 | PR-11059 |
| A-TKL1-E | A-PGK1p-TKL1-CYC1t-E | BB0947 | PR-7587 | PR-11059 |
| A-TKL1-B | B-PGK1p-TKL1-CYC1t-C | BB0947 | PR-7587 | PR-7588 |
| A-ARO4^K229^L-E | A-TEF1p-ARO4K229L-NAT5t-E | BB0622+BB0919+BB0625 | PR-7587 | PR-11059 |
| B-ARO4^K229L^-C | C-TEF1p-ARO4K229L-NAT5t-F | BB0622+BB0919+BB0625 | PR-7589 | PR-7590 |
| A-ARO1^∆aroE^-E | A-TPI1p-ARO1^∆aroE^-ADH1t-E | BB0925+BB0920+BB0623 | PR-7587 | PR-11059 |
| C-ARO1^∆aroE^-F | F-TPI1p-ARO1^∆aroE^-ADH1t-E | BB0925+BB0920+BB0623 | PR-7591 | PR-8276 |
| **Other biobricks** | | | | |
| BB10 | PGK1 promoter | gDNA *S. cerevisiae* (CEN.PK113-7D) | PR-7 | PR-8 |
| BB11 | double promoters TRF1p-PGK1p | gDNA *S. cerevisiae* (CEN.PK113-7D) | PR-6 | PR-8 |
| BB110 | *AGO1* from *N. castellii* | gDNA*N. Castellii* | PR-496 | PR-497 |
| BB111 | *DCR1* from *N. Castellii* | gDNA *N. castellii* | PR-498 | PR-499 |
| BB263 | ScTKL1 | gDNA *S. cerevisiae* (CEN.PK113-7D) | PR-1401 | PR-1400 |
| BB264 | ScTAL1 | gDNA *S. cerevisiae* (CEN.PK113-7D) | PR-1403 | PR-1402 |
| BB469 | RFP | pCfB394 | PR-3111 | PR-3112 |
| BB707 | CFP | pCfB396 | PR-8493 | PR-8494 |

**Supplementary Table S5. List of plasmids**

| **Plasmid** | **Description** | **Parent vector** | **BioBricks** | **Reference** |
| --- | --- | --- | --- | --- |
| pCfB47 | pX-3-DR-*KlURA3-ccdB* |  |  | (Jensen *et al.* 2014) |
| pCfB61 | pSH65: *CEN/ARS pGAL1*-*Cre*; ble^R^ |  |  | Euroscarf |
| pCfB255 | pX-2-loxP-*KlURA3* |  |  | (Jensen *et al.* 2014) |
| pCfB257 | pX-3-loxP-*KlLEU2* |  |  | (Jensen *et al.* 2014) |
| pCfB258 | pX-4-loxP-*SpHIS5* |  |  | (Jensen *et al.* 2014) |
| pCfB284 | pX-3-*KlURA3-AGO1*<-T*EF1p*-*PGK1p*->*DCR1* | pCfB47 | BB010, BB0110, BB0111 | This study |
| pCfB388 | pXI-1-loxP-*KlLEU2* |  |  | (Jensen *et al.* 2014) |
| pCfB393 | pX-2-loxP-*KlURA3*-*CFP*<-*TEF1p* |  |  | (Jensen *et al.* 2014) |
| pCfB394 | pX-3-loxP-*KlLEU2*-*RFP*<-*TEF1p* |  |  | (Jensen *et al.* 2014) |
| pCfB395 | pX-4-loxP*SpHis5*-*YFP*<-*TEF1p* |  |  | (Jensen *et al.* 2014) |
| pCfB1048 | pX-3-KlLEU2-*TAL1*-*TKL1* | pCfB257 | BB010, BB0263, BB0264 | This study |
| pCfB1238 | pX-2-loxP-*KanMX*-*ARO4^K229L^*<-*TDH3p*-*TEF1p*->*ARO1^∆aroE^* |  |  | (Skjoedt *et al.* 2016) |
| pCfB1239 | pXI-1-loxP-*KlLEU2*-*PaAroZ*<-*TDH3p*-*TEF1p*->*CaCatA* |  |  | (Skjoedt *et al.* 2016) |
| pCfB1241 | pTY4-*KlURA3*-*KpAroY.B*<-*TDH3p*-*TEF1p*->*KpAroY.Ciso* |  |  | (Skjoedt *et al.* 2016) |
| pCfB1767 | pRS414-*TRP1*-*TEF1p*-*Cas9*-*CYC1t* |  |  | (DiCarlo *et al.* 2013) |
| pCfB1791 | pTAJAK1; 2m-*CAN1*.Y gRNA-*KlLEU2* |  |  | (Ronda *et al.* 2015) |
| pCfB2310 | pgRNA-SNR52p-gRNA.CAN1-SUP4t_*NatMx* |  |  | (Stovicek, Borodina and Forster 2015) |
| pCfB2312 | pRS414-*KanMx*-*TEF1p*-*Cas9*-*CYC1t* |  |  | (Stovicek, Borodina and Forster 2015) |
| pCfB2586 | pXI-1-*KlLEU2*-*RFP*<-*TEF1p*-*PGK1p*->*CFP* | pCfB388 | BB010, BB0469, BB0707 | This study |
| pCfB2695 | pX-4-loxP-*SpHIS5*-*TEF1p*->*KpAroY.D* |  |  | (Skjoedt *et al.* 2016) |
| pCfB3034 | pX-3-MarkerFree |  |  | (Jessop-Fabre *et al.* 2016) |
| pCfB3041 | pgRNA-*NatMx*-X-3 |  |  | (Jessop-Fabre *et al.* 2016) |
| pCfB3100 | pX-3-*AGO1*<-*TEF1p*-*PGK1p*->*DCR1* | pCfB3034 | BB010, BB0110, BB0111 | This study |

**References**

DiCarlo JE, Norville JE, Mali P *et al.*Genome engineering in *Saccharomyces cerevisiae* using CRISPR-Cas systems. *Nucleic Acids Res* 2013;**41**:4336–43.

Jensen NB, Strucko T, Kildegaard KR *et al.*EasyClone: method for iterative chromosomal integration of multiple genes in *Saccharomyces cerevisiae*. *FEMS Yeast Res* 2014;**14**:238–48.

Jessop-Fabre MM, Jakočiūnas T, Stovicek V *et al.*EasyClone-MarkerFree: A vector toolkit for marker-less integration of genes into *Saccharomyces cerevisiae* via CRISPR-Cas9. *Biotechnol J* 2016;**11**:1110–7.

Ronda C, Maury J, Jakočiunas T *et al.*CrEdit: CRISPR mediated multi-loci gene integration in *Saccharomyces cerevisiae*. *Microb Cell Factories* 2015;**14**:97.

Skjoedt ML, Snoek T, Kildegaard KR *et al.*Engineering prokaryotic transcriptional activators as metabolite biosensors in yeast. *Nat Chem Biol* 2016;**12**:951-958. DOI: 10.1038/nchembio.2177.

Stovicek V, Borodina I, Forster J. CRISPR–Cas system enables fast and simple genome editing of industrial *Saccharomyces cerevisiae* strains. *Metab Eng Commun* 2015;**2**:13–22.
